# High-speed photoacoustic-guided wavefront shaping for focusing light in scattering media

**DOI:** 10.1364/OL.412572

**Published:** 2021-03-01

**Authors:** Tianrui Zhao, Sebastien Ourselin, Tom Vercauteren, Wenfeng Xia

**Affiliations:** School of Biomedical Engineering and Imaging Sciences, King’s College London, 4th Floor, Lambeth Wing, St Thomas’ Hospital, London SE1 7EH, UK

## Abstract

Wavefront shaping is becoming increasingly attractive as it promises to enable various biomedical applications by breaking through the optical diffusion limit that prevents light focusing at depths larger than ∼1mm in biological tissue. However, despite recent advancements in wavefront shaping technology, such as those exploiting non-invasive photoacoustic-guidance, *in vivo* demonstrations remain challenging mainly due to rapid tissue speckle decorrelation. In this work, we report a high-speed photoacoustic-guided wavefront shaping method with a relatively simple experimental setup, based on the characterization of a scattering medium with a real-valued intensity transmission matrix. We demonstrated light focusing through an optical diffuser by optimizing 4096 binary amplitude modulation modes of a digital micromirror device within ∼300ms, leading to a system runtime of 75 µs per input mode, which is 3 orders of magnitude smaller than the smallest runtime reported in literature so far using photoacoustic-guided wavefront shaping. Thus, our method is a solid step forward toward *in vivo* applications of wavefront shaping.

Focusing light through biological tissue is of great significance for applications in a broad spectrum of life sciences including deep tissue optical microscopy, optogenetics, micro-manipulation, and laser microsurgery. Over the last decade, wavefront shaping (WS) emerged as an effective approach for focusing light at target positions inside or through scattering media [1–4]. The basic principle of WS is to spatially modulate the incident light field to match the microscopic scattering properties of the medium so that the scattering-induced phase distortion is corrected. In this way, the scattered waves interfere constructively at the target position behind a scattering layer to form a sharp focus. Conventional methods for optical WS usually require a detector to be placed behind the scattering medium to measure the light intensity at the target position, which restricts their practical uses in biological tissues [5–7]. To address this issue, various non-invasive methods were studied that provide feedback with internal “guide-stars” so that the light intensity at the target location can be maximized. These guide-stars include exogenous fluorescence particles [8–11], ultrasound (US)-tagged light based on acousto-optics [12–14], and light-generated US waves based on the photoacoustic (PA) effect [15–21].

PA-guided WS has attracted significant research interest in recent years since it avoids the use of a complex optical reference arm as those in phase conjugation-based WS or exogenous particles as guide-stars. It could potentially allow for a relatively large penetration depth as acoustic attenuation is much smaller than optical attenuation in tissue. With PA-guided WS, a focused US transducer measures the light-generated US/PA waves at the acoustic focus. By iteratively optimizing the wavefront of the incident light, the intensity of the acoustic pressure is maximized, leading to an intense optical focus with its size defined by the focus size of the US transducer. The group of Chen *et al.* [15] was the first to demonstrate this concept with a deformable mirror. Liquid-crystal spatial light modulators (LC-SLMs) and digital micromirror devices (DMDs) were also used by other groups [18,19,22]. Several methods were proposed to further reduce the size of the optical focal spot including the use of the spatially non-uniform sensitivity of the US transducer [17], non-linear PA signals induced by the Grüneisen relaxation effect [16], and PA amplitude fluctuations from flowing particles [21]. However, although these methods have proven effective for light focusing, it usually takes several minutes to several hours to form a light focus due to the use of time-consuming iterative algorithms and low-repetition-rate lasers and spatial light modulators (SLMs) [16–22]. In consideration of the rapid speckle decorrelation in dynamic tissues [1,5,13], a high-speed approach enabling light focusing with PA-guidance is highly desired for *in vivo* applications.

In our previous work, we reported that a real-valued intensity transmission matrix (RVITM) can be used to approximately connect the input and output light intensities of a disordered medium (e.g., multimode optical fiber), and with which input binary and gray scale images can be retrieved from measured intensities of the output speckles [23]. In this study, we further demonstrate that the RVITM can also be used for focusing light through scattering media as it encodes both the phase and amplitude information of a corresponding complex-valued transmission matrix (TM). Different from conventional methods that optimize the PA signal via iterative algorithms, our method relies on a high-speed characterization of the scattering medium with a RVITM, and directly calculates an optimal light input pattern based on the encoded phases information in the RVITM for focusing.

The RVITM-based light focusing procedure comprised a characterization step and a focusing step. In the characterization step, a Hadamard matrix H∈ (−1, +1) with dimensions of N×N was used to construct two binary matrices $H_{1}=(H+1)/2$ and $H_{2}=(-H+1)/2$. Each column of a binary matrix, $\left[H_{1},H_{2}\right]$, was then converted to a square matrix that was used to spatially modulate the incident laser beam onto the scattering medium using the DMD, while the corresponding PA waves were recorded by a focused US transducer. Based on the principle of PA signal generation [24,25], the amplitude of the received US signal with the $k^{\text{th}}$ input DMD pattern can be expressed as $P^{k}=aS\Gamma \mu_{a}F^{k}$, where a is a constant account for the attenuation loss during US propagation, S is the sensitivity of the US detector, Γ is the Grüneisen coefficient, $\mu_{a}$ is the optical absorption coefficient, and $F^{k}$ is the local optical fluence, with $F^{k}=\frac{\int I^{k^{\prime }}dt}{A}$, where $\int I^{k^{\prime }}dt$ is the integration of the light intensity, $I^{k^{\prime }}$, over the duration of the light pulse when the $k^{\text{th}}$ DMD pattern is displayed, and A is the illumination area. Since the pulse duration, T, is constant, $F^{k}$ can be further expressed as $F^{k}=\frac{I^{k}T}{A}$, where $I^{k}$ is the average light intensity over T, and we have $P^{k}=\frac{aS\Gamma \mu_{a}I^{k}T}{A}$, where $\frac{aS\Gamma \mu_{a}T}{A}$ is a constant under the experimental condition. For simplicity, we define $\alpha =\frac{aS\Gamma \mu_{a}T}{A}$. As such, $I^{k}$ is linearly proportional to $P^{k}$ and thus maximizing the amplitude of $P^{k}$ is equivalent to focusing light at the transducer focus. With the approximate relationship between the intensities of the input and output light field through a scattering medium that can be connected by an RVITM [23], $I^{k}$ and $\left[H_{1},H_{2}\right]$ can be approximately connected by a row of the RVITM elements, ${RVITM}_{r}$, that correspond to light transport from all the DMD input positions to the target location (US transducer focus) as

(1) $$\begin{array}{ll} \left[I^{1},I^{2},\cdots ,I^{2N}\right] & =\frac{1}{\alpha }[P^{1},P^{2},\cdots ,P^{2N}]\\ & ={RVITM}_{r}∙[H_{1},H_{2}]. \end{array}$$

As ${\left[H,-H\right]}^{T}={\left[H,-H\right]}^{-1}$ owing to the Hadamard matrix properties, we have

(2) $$\begin{array}{ll} {RVITM}_{\text{PA}} & =\left[\begin{array}{c} 2P^{1}-P^{1},2P^{2}-P^{1},\cdots ,2P^{2N}-P^{1} \end{array}\right]\;\;\;\\ & ∙{\left[H,-H\right]}^{T}, \end{array}$$

where we define ${RVITM}_{\text{PA}}=\alpha {RVITM}_{r}$, which connects the light intensities from all the input positions to the PA signal amplitude at the target location. So, a positive ${RVITM}_{\text{PA}}$ value means that the corresponding micromirror contributes positively to the optical intensity. Thus, in the focusing step, an optimal DMD pattern was determined by switching “ON” all the DMD micromirrors that corresponded to positive ${RVITM}_{\text{PA}}$ values for focusing light at the target location.

To further understand the physics behind ${RVITM}_{\text{PA}}$, the matrix element ${RVITM}_{\text{PA}}^{n}$ connecting the light input at the $n^{\text{th}}$ micromirror and the output PA signal amplitude at the acoustic focus is expressed as

(3) $$\begin{array}{c} \begin{array}{c} {RVITM}_{\text{PA}}^{n}=2\sum_{k=1}^{2N}\;(P^{k}-P^{1})h_{n}^{k}=2\sum_{k=1}^{2N}P^{k}h_{n}^{k}=2\alpha \sum_{k=1}^{2N}I^{k}h_{n}^{k} \end{array}\;, \end{array}$$

where $h_{n}^{k}$ is the element from [H,−H] corresponding to the $n^{\text{th}}$ micromirror position in the $k^{\text{th}}$ DMD pattern as $h_{n}^{k}=2E_{n}^{k}-1$, and N is the total number of independent micromirrors. The light intensity at the target output location corresponding to the $k^{\text{th}}$ DMD pattern, $I^{k}$, can be expressed as $I^{k}=|\sum_{n=1}^{N}t_{n}E_{n}^{k}{|}^{2}$ based on the conventional TM theory with $E_{n}^{k}\in (0,1)$ representing the light field at the $n^{\text{th}}$ input micromirror position in the $k^{\text{th}}$ pattern and $t_{n}$ representing the corresponding complex-valued transmission constant [1]. Then, as ${RVITM}_{\text{PA}}^{n}$ is the sum of the output over the total number of 2N input DMD patterns ($2\alpha |\sum_{n=1}^{N}t_{n}E_{n}^{k}{|}^{2}h_{n}^{k}$), we have

(4) $$\begin{array}{ll} {RVITM}_{PA}^{n} & =2\alpha [|t_{n}{|}^{2}\sum_{k=1}^{2N}{\left(E_{n}^{k}\right)}^{2}h_{n}^{k}+\sum_{i=1,i\neq n}^{N}|t_{i}{|}^{2}\sum_{k=1}^{2N}|E_{i}^{k}{|}^{2}h_{n}^{k}\\ & \;+\sum_{i=1,i\neq n}^{N}(t_{n}t_{i}^{*}+t_{n}^{*}t_{i})\sum_{k=1}^{2N}(E_{n}^{k}E_{i}^{k})h_{n}^{k}\\ & \;+\sum_{i=1,i\neq n}^{N}\sum_{j=2,j\neq n,j>i}^{N}(t_{i}t_{j}^{*}+t_{i}^{*}t_{j})\sum_{k=1}^{2N}(E_{i}^{k}E_{j}^{k})h_{n}^{k}]. \end{array}$$

According to the properties of the Hadamard matrix, during the characterization step, every micromirror in the DMD is switched “ON” or “OFF” for N times. With the total number of N patterns where the $n^{\text{th}}$ micromirror is switched “ON” ($h_{n}^{k}=1$, $E_{n}^{k}=1$), the number of times for every other micromirror to be switched “ON” ($E_{i}^{k}=1$) is N/2, and that for every combination of other two micromirrors to be switched “ON” ($E_{i}^{k}=1$, $E_{j}^{k}=1$) simultaneously is N/4. Similarly, when the $n^{\text{th}}$ micromirror is switched “OFF” ($h_{n}^{k}=-1$, $E_{n}^{k}=0$), the $i^{\text{th}}$ micromirror is switched “ON” for N/2 times while the $i^{\text{th}}$ and the $j^{\text{th}}$ micromirrors are simultaneously switched “ON” for N/4 times. As a result, $\sum_{k=1}^{2N}{\left(E_{n}^{k}\right)}^{2}h_{n}^{k}=N$, $\sum_{k=1}^{2N}|E_{i}^{k}{|}^{2}h_{n}^{k}=0$, $\sum_{k=1}^{2N}(E_{n}^{k}E_{i}^{k})h_{n}^{k}=N/2$, and $\sum_{k=1}^{2N}(E_{i}^{k}E_{j}^{k})h_{n}^{k}=0$; therefore, ${RVITM}_{\text{PA}}^{n}$ can be further expressed as

(5) $$\begin{array}{ll} {RVITM}_{PA}^{n} & =2\alpha [N|t_{n}{|}^{2}+\frac{N}{2}\sum_{i=1,i\neq n}^{N}(t_{n}t_{i}^{*}+t_{n}^{*}t_{i})]\\ & =\alpha N[t_{n}t_{n}^{*}+t_{n}^{*}t_{n}+\sum_{i=1,i\neq n}^{N}(t_{n}t_{i}^{*}+t_{n}^{*}t_{i})]\\ & =\alpha N(t_{n}R^{*}+t_{n}^{*}R)\\ & =\alpha NA_{R}A_{n}\cos(\theta_{n}-\phi_{R}), \end{array}$$

where * denotes the complex conjugate operator, R is the output light field at the target position when all the micromirrors are switched “ON,” $\phi_{R}$ and $A_{R}$ are the phase and amplitude of R, and $\theta_{n}$ and $A_{n}$ are the phase and amplitude of $t_{n}$, respectively. According to Eq. (5), when the phase $\theta_{n}$ is within the range of [$\phi_{R}-\pi /2,\phi_{R}+\pi /2$], the value of ${RVITM}_{\text{PA}}^{n}$ is positive, and therefore, switching “ON” those corresponding micromirrors leads to constructive light interference at the target output position for focusing.

To demonstrate the feasibility of the RVITM-based WS method, PA-guided light focusing through an optical diffuser was performed as illustrated in Fig. 1. A pulsed laser emitting at 532 nm with a pulse duration of 2 ns (SPOT-10-200-532, Elforlight, Daventry, UK) was used as the light source for PA signal excitation. A DMD (DLP7000, 768×1080 pixels, Texas Instruments, Texas) was used to project incident light beam onto the diffuser (N-BK7 Ground Glass Diffuser, 220 Grit, Thorlabs, New Jersey, USA) with a modulated wavefront via a convex lens (AC254-030-A-ML, Thorlabs, New Jersey). The DMD was operated in an uninterrupted mode at 47 kHz. The number of input modes used for light modulation was 64×64, with 2×2 micromirrors grouped (switch “ON” or “OFF” at the same time) as one independent input mode. A piece of black tape was placed ∼5mm behind the diffuser (situated at the focal distance from the US transducer) as an optical absorber. A flat single-element piezoelectric transducer (V358, central frequency, 50 MHz; diameter, 6.35 mm; Olympus, Japan) was used to detect the generated US signals with a silica plano–concave lens (LC4210; f=−25mm; Thorlabs, New Jersey, USA) attached on its active surface for acoustic focusing. Both the optical absorber and the US transducer were immersed in water for acoustic coupling. The generated acoustic waves acquired by the US transducer were amplified (SPA.1411, Spectrum Instrumentation, Grosshansdorf, Germany), digitized by a data acquisition card (DAQ) (M4i.4420, Spectrum Instrumentation, Grosshansdorf, Germany) and transferred to a PC (Intel i7, 3.2 GHz) for processing. Synchronization between the laser pulsing and DMD display was provided by an arbitrary waveform generator (33600A, Keysight, Santa Rosa, California). After the implementation of light focusing, the absorber and the transducer were replaced with a camera and a convex lens (AC254-050-A-ML, Thorlabs, New Jersey) to capture the optical speckle patterns at the focal plane of the US transducer.

**Fig. 1.**
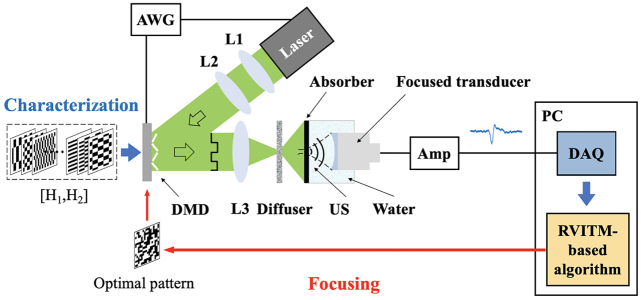
Schematic illustration of the experimental setup and principle. AWG, arbitrary waveform generator; Amp, amplifier; L1–L3, convex lenses.

Experimental results are shown in Fig. 2. With the optimal DMD pattern, the PA signal amplitude increased by 6.67 times compared to that with a random DMD pattern [Fig. 2(a)]. The corresponding optical speckle pattern with a random DMD pattern spread out across a region approximately 320µm×320µm onto the absorber location [Fig. 2(d)]. When the optimal DMD pattern was displayed, the light energy was concentrated on a smaller region with saturated pixels [Fig. 2(e)]. After reducing the light intensity with a neutral density filter, the light focusing effect was clearly visualized in Fig. 2(f). Figures 2(c) and 2(d) show the lateral and vertical intensity profiles across the centers of the global maxima of the speckle patterns (along the dashed lines) in Figs. 2(d) and 2(f). The dimensions of the optical focus, indicated by the full width at half-maximum values of the Gaussian fits to the intensity profiles with the optimal DMD pattern, were (∼56µm×40µm), which were consistent to the diameter of the US transducer’s focus (∼49µm). A light enhancement factor of 6.89 was achieved by calculating the ratio of the average values of the image intensities in the focal region after and before focusing.

**Fig. 2.**
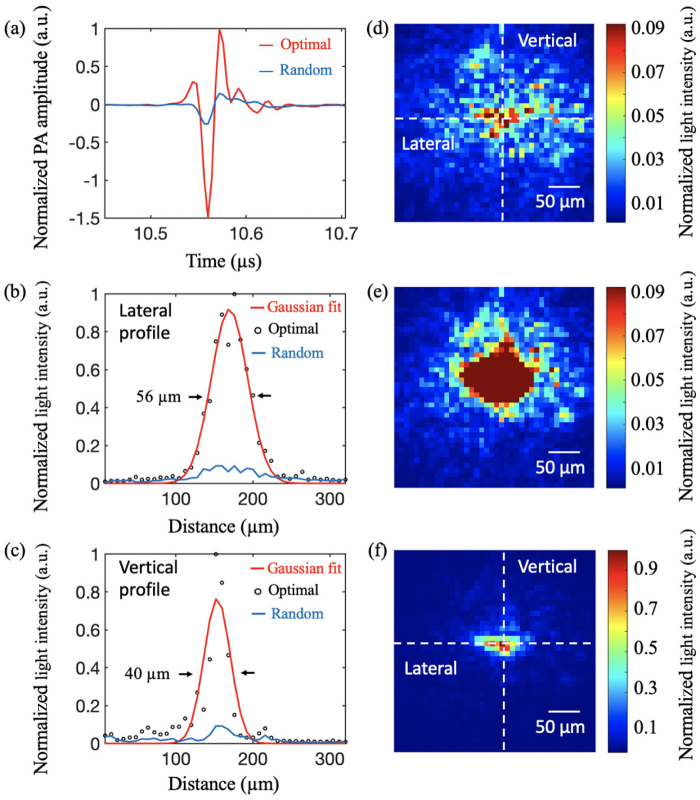
Focusing light through an optical diffuser. (a) Comparison of photoacoustic (PA) signals generated with random DMD patterns and an optimal DMD pattern. The blue line is an average of 64 PA signals acquired when 64 random DMD patterns were displayed, and the red line is an average of 64 PA signals when an optimal DMD pattern was displayed. (b) Lateral and (c) vertical intensity profiles along the dashed lines in the captured optical speckle patterns at the absorber plane when (d) a random DMD pattern and (e) an optical pattern were displayed. (f) The same optical speckle pattern as in (e) when a neutral density filter was used to attenuate the light intensity to avoid overexposure. To facilitate comparison, the light intensity values in (f) have taken into account the light attenuation by the filter. a.u., arbitrary unit.

The time cost of each step in the workflow of the RVITM-based method is shown in Fig. 3. Before the characterization step, 8192 input DMD patterns ([$H_{1},H_{2}$]) were uploaded onto the memory of the DMD. The largest time cost was for the display of these DMD patterns at a rate of 47 kHz (175 ms), while PA signals were acquired at the same time. It took another 66 ms to transfer the PA signals from the DAQ card to PC memory. The amount of time used for calculating ${RVITM}_{\text{PA}}$ values and producing an optimal DMD pattern was only 58 ms using a custom script implemented in MATLAB. The final step for uploading and displaying the optimal pattern on the DMD for light focusing took 7 ms. Thus, the total system runtime time was 306 ms.

**Fig. 3.**
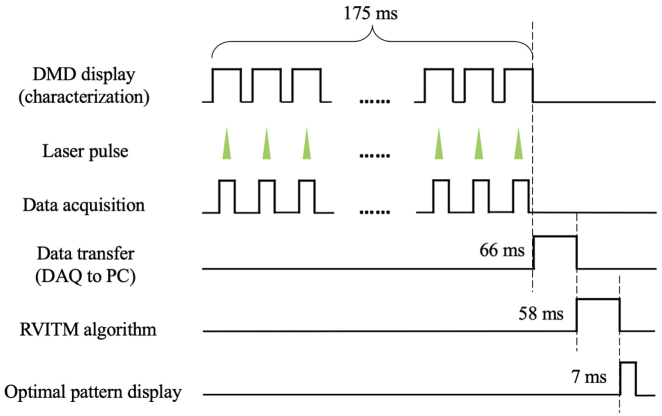
Workflow of the RVITM-based wavefront shaping for focusing light through scattering media. The total system runtime is 306 ms, and the total number of input DMD modes is 4096. DAQ, data acquisition.

The system runtime for optimizing each input mode is 75 µs. This is a significant improvement in speed for WS with a PA guide-star. In comparison, the shortest runtime in previous studies was 0.7 s per input mode with an enhancement of 60, achieved by optimizing 20736 LC-SLM modes [16]. Our non-iterative method directly calculates a RVITM from the characterization measurements and produces an optimal DMD pattern accordingly, which dramatically shortens the time scale for light focusing. By investigating the mathematical expression of ${RVITM}_{\text{PA}}$ based on conventional TM theory, we further proved that ${RVITM}_{\text{PA}}$ encodes both phase and amplitude information of the light field changes from the DMD to the acoustic focus into real-valued transmission elements, and therefore, an optimal DMD pattern can be easily obtained by switching “ON” the micromirrors with positive ${RVITM}_{\text{PA}}$ values that contributed positively to light focusing at the target location. The time costs for data transfer and ${RVITM}_{\text{PA}}^{n}$ calculation could be further reduced by using a field programmable gate array (FPGA) board [7]. However, achieving a total runtime that is on the order of ms suitable for *in vivo* applications requires further technological advancement of SLMs that provide faster modulations.

The light enhancement within the acoustic focal region (6.89) is comparable to those obtained from other studies using a linear PA signal as feedback [18,20,21]. The theoretical enhancement factor can be expressed as $\eta =\frac{N}{2\pi M}$ [5], where M is the number of output modes within the acoustic focus. In our case, a theoretical enhancement was calculated as 14.5 (M was estimated as ∼45). The lower than expected enhancement performance could be attributed to the system instability, the fluctuation of laser mode and energy, the non-uniformity of the laser beam, and the noise of the acoustic signals. To further improve the enhancement, a larger N could be used. However, this will result in a larger number of patterns for display. Although the characterization and data transfer time scales linearly with N, the dependency of the data processing time on N requires further investigation. Compared to other non-invasive methods such as those using fluorescence signals as feedback, WS with PA guide-stars relies on the intrinsic optical absorption of tissue and, thus, avoids the use of exogenous particles, and it promises to provide a larger focusing depth as acoustic waves suffer from much less attenuation in tissue than light. However, as the focusing depth increases, the speckle grain size reduces, leading to an increased M. This will reduce the expected performance (e.g., SNR) of our method among others. Phase conjugation with ultrasonically encoded light as feedback has been demonstrated with a focusing speed of 5.6 ms [13], on the order of the tissue decorrelation time. However, the attained focal spot size was restricted by the acoustic focus. In the future, the feasibility of generating a sub-acoustic optical focus with the RVITM-based PA-guided WS method will be studied by using a dual-pulse PA excitation scheme similar to that used in Ref. [16]. With a reduced focal spot size, the light enhancement could be significantly improved as demonstrated in Refs. [16,21]. The position of the focus was determined by the focus of the US detector, which could be translated for focusing into other locations. Alternatively, scanning of the focus could be performed based on the memory effect [19].

In summary, we developed a high-speed method to focus light through scattering media with a PA guide-star. By employing a non-iterative algorithm and binary amplitude modulations with a DMD, we demonstrated the feasibility to focus light through an optical diffuser to a spot of ∼56µm×40µm within a total system runtime of 306 ms. With further improvement in speed, this method could be useful in various non-invasive applications such as microscopy, optogenetics, micro-manipulation, and microsurgery.
